# Empirical evidence for metabolic drift in plant and algal lipid biosynthesis pathways

**DOI:** 10.3389/fpls.2024.1339132

**Published:** 2024-01-31

**Authors:** Maëlle Zonnequin, Arnaud Belcour, Ludovic Delage, Anne Siegel, Samuel Blanquart, Catherine Leblanc, Gabriel V. Markov

**Affiliations:** ^1^ Sorbonne Université, CNRS, Integrative Biology of Marine Models (LBI2M, UMR8227), Station Biologique de Roscoff (SBR), Roscoff, France; ^2^ Univ Rennes, Inria, CNRS, IRISA, Equipe Dyliss, Rennes, France; ^3^ Univ. Grenoble Alpes, Inria, Grenoble, France

**Keywords:** metabolic pathway evolution, oxylipins, sterols, phosphatidyl-choline, biotic interactions

## Abstract

Metabolic pathway drift has been formulated as a general principle to help in the interpretation of comparative analyses between biosynthesis pathways. Indeed, such analyses often indicate substantial differences, even in widespread pathways that are sometimes believed to be conserved. Here, our purpose is to check how much this interpretation fits to empirical data gathered in the field of plant and algal biosynthesis pathways. After examining several examples representative of the diversity of lipid biosynthesis pathways, we explain why it is important to compare closely related species to gain a better understanding of this phenomenon. Furthermore, this comparative approach brings us to the question of how much biotic interactions are responsible for shaping this metabolic plasticity. We end up introducing some model systems that may be promising for further exploration of this question.

## Introduction

The comparison of metabolic and signaling pathways across organisms often leads to noticing similarities and differences among them, as well illustrated by the keynote lecture on N-acylethanolamines at the virtual ISPL2020 (Cannon and Chapman, 2021). However, going from specific examples to general principles is difficult, because it necessitates to find a balance between deep analysis of specific examples, and broad overview that implies a certain level of simplification and abstraction. The concept of metabolic pathway drift was introduced by some of us to formalize ideas that emerged from our analysis of specific pathways (Belcour et al., 2020). Here the purpose is to challenge this concept beyond our own experimental case studies, in order to check its robustness to other experimental data and to further discuss a theoretical framework that we hope could be also helpful for other researchers interested in metabolic pathway evolution.An important research topic in developmental and evolutionary biology has been to understand the homology of morphological features between species. Especially how conserved were the molecular pathways leading to the development of these features? Indeed, one could expect that similar molecular pathways produce similar morphological features. But it has been shown that developmental pathways could diverge through time without impact on the outcome of the morphological feature. These changes seem to be determined not by natural selection (as the phenotypic outcome is not modified) but by chance. This led to the definition of the Developmental System Drift (True and Haag, 2001), later acknowledging that similar ideas came from the concept of balancing selection (Haag and True, 2021). This drift explains how similar morphological structures in different species can be maintained even if the molecular mechanisms underlying their formations undergo variations. Another description of Developmental system drift was also made in gene expression of molar development in rodents (Sémon et al., 2020). Upper molars in rodents show a drastic change in morphology, whereas the lower molars display little morphological variations. However, the authors found that gene expressions display a lot of variation across rodents in both upper and lower molars during molar development. So despite a similar structure in lower molars shared across rodents, developmental gene expression of these teeth shows variation.

**Figure 1:**
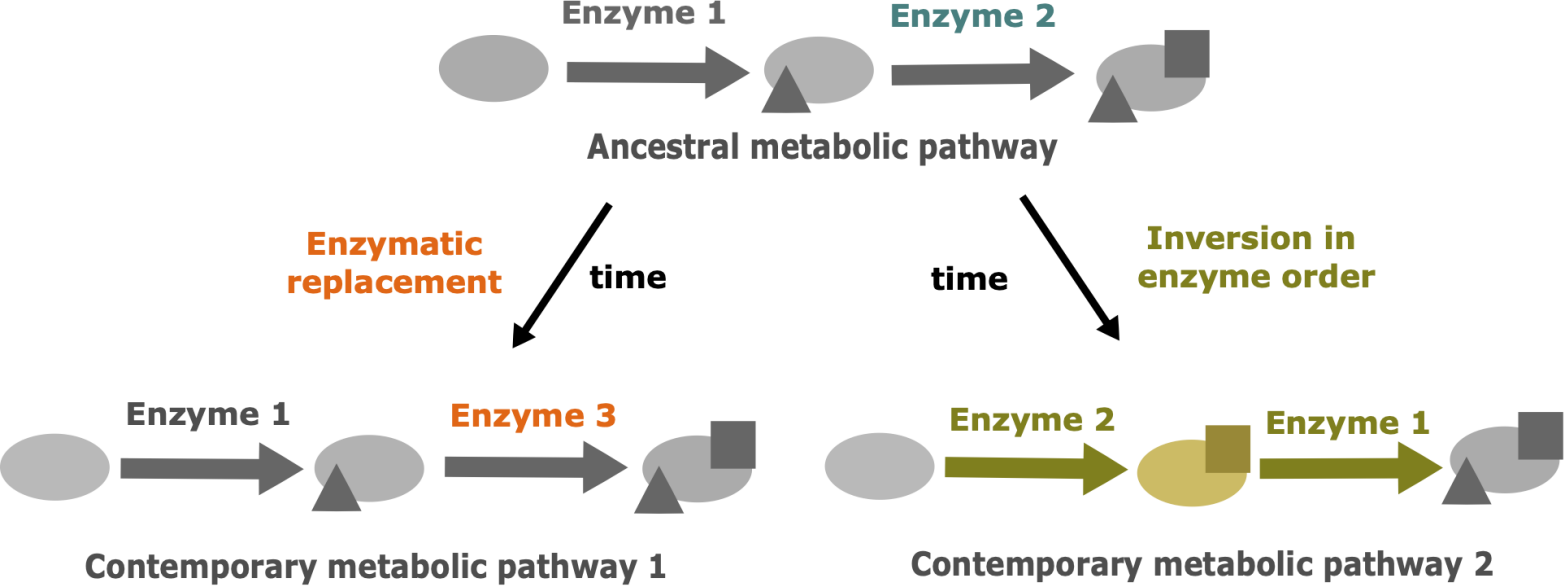
The two main drift mechanisms leading to the divergence of metabolic pathways despite the conservation of the initial precursor and the final product. Modified from Belcour et al., 2020. The triangles represent the result of metabolic transformations catalyzed by enzyme 1 and the squares represent the result of metabolic transformations catalyzed by enzymes 2 or 3. Blue: ancestral enzyme 2. Orange: drift by enzymatic replacement of enzyme 2 by enzyme 3. Yellow: drift by inversion in enzyme order, leading to a new intermediate metabolite.

Similar drift phenomenons have been described in other fields, such as protein evolution (Hart et al., 2014). In this article the authors explored the thermodynamic system drift of the protein ribonuclease H1 from *Thermus thermophilus* and *Escherichia coli*. In particular, they proposed that the melting temperature is under selection and the underlying mechanisms of thermostability could vary without impacting the associated phenotype. Other cellular processes involving proteins could be impacted by these drifts (Palsson and Steele, 2023). A major one is the metabolism with enzymes catalyzing biochemical reactions. In metabolism, multiple metabolic pathways are shared between species. Many variations can occur in these metabolic pathways. Thus, we proposed that drift can also occur in metabolic pathways (**Figure 1**). Metabolic pathways of related organisms can produce the same output metabolite from the same input metabolite. This metabolic “phenotype” is under selective pressure and remains conserved. However, changes in the cascade of biochemical reactions and intermediate products can be neutral and subject to evolutionary drift.

## Empirical evidence: what is known and what remains to be demonstrated

### An alternative epoxy alcohol synthase in diatoms, suggesting an enzymatic replacement

**Figure 2:**
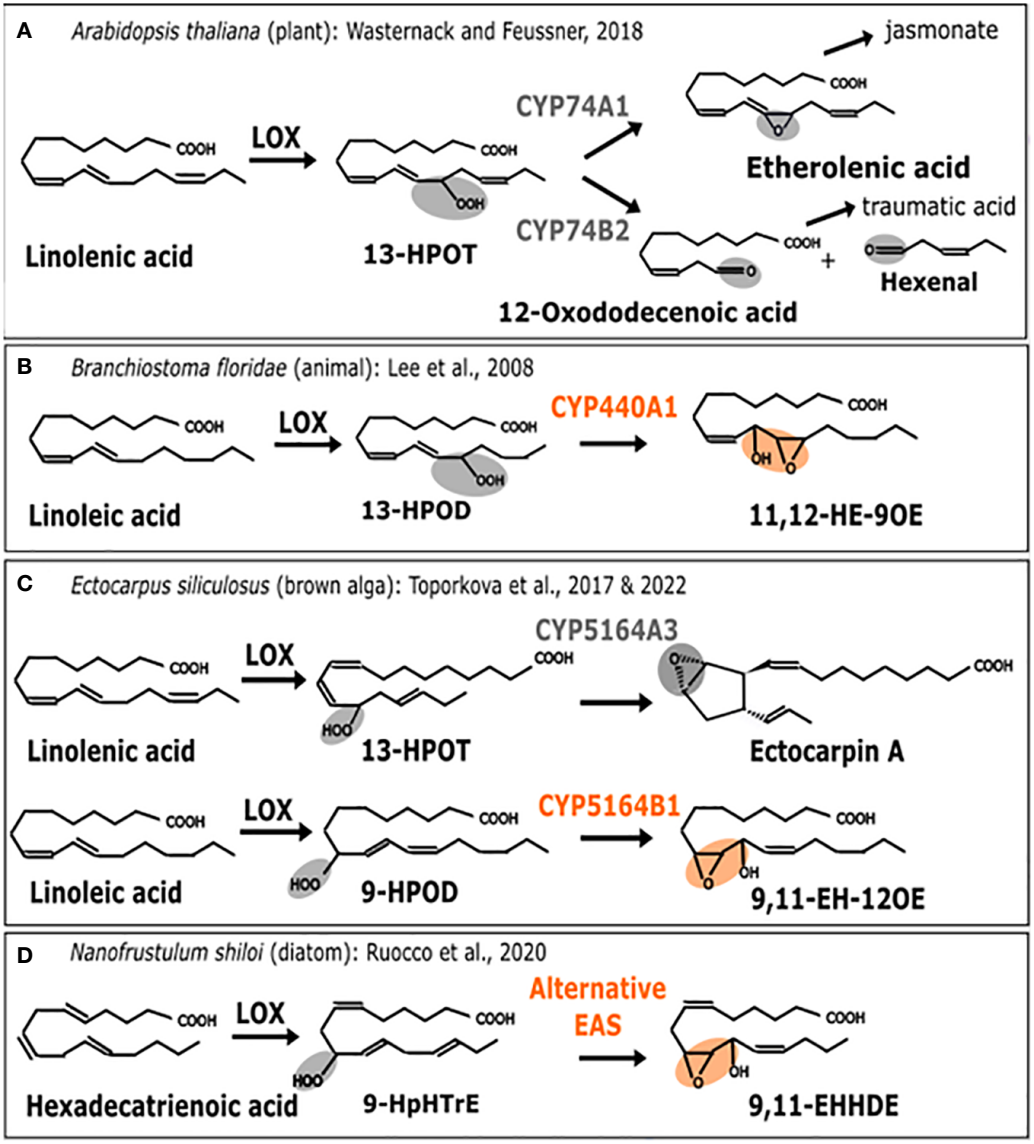
Enzymatic replacement in the oxylipin biosynthesis pathway. Comparison of metabolic pathways for oxylipin biosynthesis. **(A)** *A*. *thaliana* (plant). 13-H(P)OT: (9Z, 11E, 13S, 15Z)-13-hydro(pero)xy-9,11,15-octadecatrienoic acid **(B)** *B*. *floridae* (animal). 13-H(P)OD: (9Z, 11E, 13S, 15Z)-13-hydro(pero)xy-9,11,15-octadecadienoic acid, 11,12-HE-9OE: 11S-hydroxy-12R,13S-eopoxy-9Z-octadecenoic acid **(C)** *E*. *siliculosus* (brown alga). 9-HPOD:(9S, 10E, 12Z)-9-hydro(pero)xy-10,12,15-octadecadienoic acid. 9,11-EH-12OE: 9-epoxy-11-hydroxy-12-octadecenoic acid. **(D)** *N. shiloi* (diatom). 9-HpHTrE: 9-Hydroperoxy-Hexatrienoic acid. 9,11-EHHDE: 9,10-epoxy-11-hydroxy-hexadienoic acid. Epoxy alcohol synthase (EAS) biochemical activities are highlighted in orange. Other activities are in gray. First, the metabolic pathway starts with a polyunsaturated fatty acid (PUFAs) which is transformed into a fatty acid hydroperoxide under the action of lipoxygenase (LOX). Then, this substrate is used by CYP74-like enzymes to be converted into epoxy alcohol or other products such as Ectocarpin A, jasmonate (JA) or polyunsaturated aldehydes like the 3-hexenal.

Oxylipins are the substrate derivatives resulting from the oxidation of polyunsaturated fatty acids (Gerwick et al., 1991). This group of biologically active compounds performs various functions in plants (Blée, 2002), animals, algae and fungi (Brodhun and Feussner, 2011) such as responses to environmental stresses or regulating development. In particular, they can be induced in the context of interactions when attacker signals are recovered or when wounding occurs. The jasmonate phytohormone is the most studied oxylipin in plants whereas in animals, it is a family of molecules named prostaglandins. While there are some well-known differences in their biosynthetic pathways, they also share common features. It is the case of the first step of this pathway, where an oxidation process generates primary hydroperoxides which are subsequently metabolized into different derivatives by action of substrate specific enzymes (**Figure 2**).

Lipoxygenases (LOX) constitute a family of dioxygenases that catalyze the oxygenation of free polyunsaturated fatty acids to produce the corresponding hydroperoxydated derivatives. Those are further metabolized, notably into epoxy alcohols by CYP74-like enzymes from the Cytochrome P450 (CYP) super-family, thus acting as epoxyalcohol synthases (EAS) (Chang et al., 1996). The wide diversity of oxylipins found in plants is produced by a group of specialized cytochromes P450 enzymes belonging to the CYP74A subfamily (Lee et al., 2008; Nelson and Werck-Reichhart, 2011), thus, allowing the production of defense-related molecules such as jasmonates (Turner et al., 2002) or traumatic acid (**Figure 2A**) to protect against various biotic and abiotic stresses. In that case, the associated activities are allene oxide synthase (AOS) catalyzed by CYP74A1 and hydroperoxide lyase (HPL) catalyzed by CYP74A2 (**Figure 2A**) (Wasternack, 2018). Enzymes that are CYP74-related are also found in marine animals such as the cephalochordate *Branchiostoma floridae*, where it is named CYP440A1 and performs epoxyalcohol synthase activity (**Figure 2B**). This enzyme has been secondarily lost in vertebrates, including mammals (Lee et al., 2008). In the brown algal model *Ectocarpus siliculosus*, belonging to Ectocarpales, the biochemical characterization of the CYP5164B1 homolog of the CYP74 clan has shown that it epoxidizes peroxidized fatty acids as illustrated in **Figure 2C** (Toporkova et al., 2017), whereas a second paralog, CYP5164A, produces ectocarpin A through hydroperoxide bicyclase activity (Toporkova et al., 2022).

Therefore, according to this literature review (illustrated in **Figure 2**), the oxylipin metabolism pathways of plants, animals and brown algae share a similar range of oxidation activities on fatty acid hydroperoxides, that are performed by homologs of CYP74. However, enzymes from the CYP74-like family have been secondarily lost in the course of evolution in diatoms (Teng et al., 2019). Other peroxidases, such as catalases, have been shown to carry out CYP74-like AOS and HPL activities in corals (Mashhadi et al., 2016). Following these findings, it has also been proposed that epoxyalcohol synthesis activity could be directly carried out by a LOX enzyme in diatoms (Fontana et al., 2007). As shown in **Figure 2D**, this could be hypothesized in some diatoms when the epoxidized diatom molecule is most similar to the one found in brown algae (Ruocco et al., 2020). This would suggest enzyme replacement, although the full demonstration by biochemical characterization of such a bifunctional LOX is still missing. For the moment, only a few LOX homologous sequences have been reported in diatoms, and although one of them is very likely involved in fatty acid peroxidation (Sabatino et al., 2022), there is no report of a fusion protein bearing a catalase-like domain coupled to a LOX domain. In conclusion, similar final products of the oxylipin biosynthesis pathway have been identified in different organisms, but in diatoms, the conversion of the epoxidized substrate is not performed by a cytochrome P450, but by a catalase-like hemoprotein, thus, suggesting the occurrence of an enzyme replacement.

### Some enzymatic replacements in the sterol biosynthesis pathway

**Figure 3:**
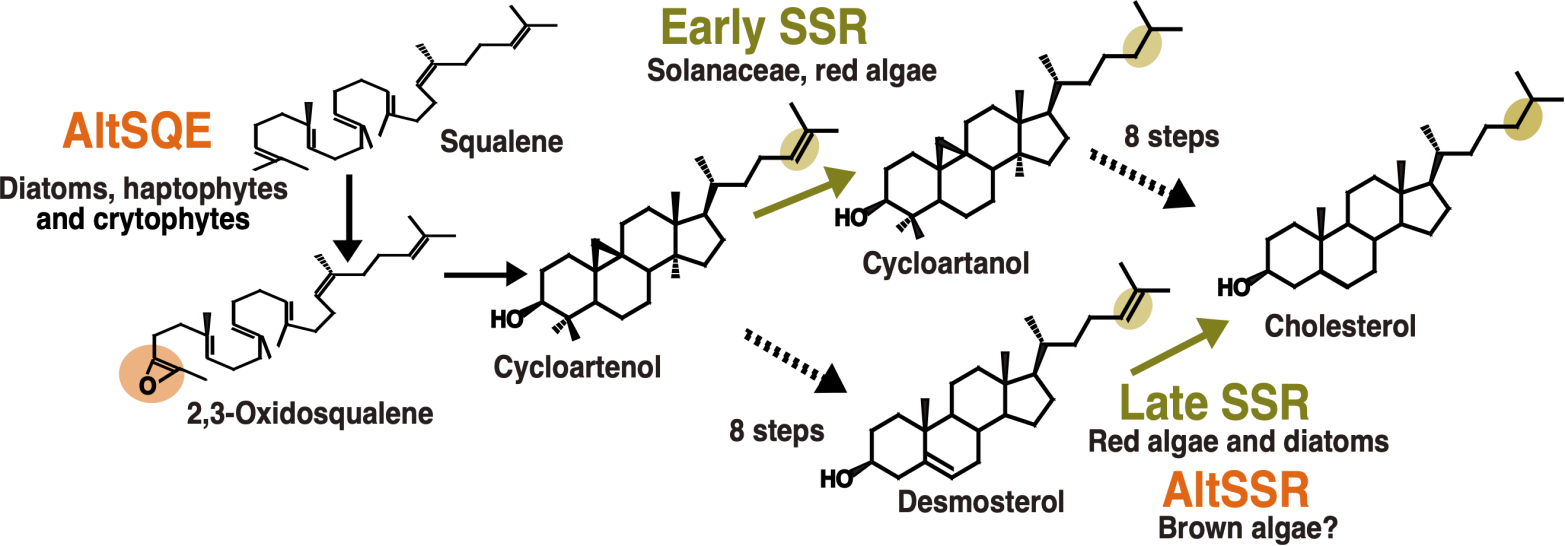
Enzymatic replacements in algal cholesterol biosynthesis pathways. In orange, reactions with demonstrated or hypothesized enzymatic replacements. In yellow, changes in enzyme order depending on biological lineages. AltSQE: alternative squalene epoxidase. AltSSR: alternative sterol side-chain reductase. Adapted from Pollier et al., 2019 and Girard et al., 2021.

The sterol biosynthesis pathway provides several examples of enzymatic replacement and, potentially also inversion in enzyme order (**Figure 3**). The most emblematic case is the replacement of the canonical squalene epoxidase (SQE), belonging to the flavoprotein monooxygenase family in most eukaryotes, by an alternative enzyme, AltSQE that belongs to the fatty acid hydroxylase protein family in diatoms, haptophytes, cryptophytes among others (Pollier et al., 2019). Initial empirical evidence came from of a lack of sensibility of *Phaeodactylum tricornutum* to an in inhibitor otherwise strongly active against yeast or plant epoxidation of squalene. Then, an alternative enzyme has been functionally identified using a mutant complementation screening with a yeast strain deficient for the canonical SQE enzyme: the sterol synthesis ability was restored by heterologous expression of AltSQEs from various eukaryotic lineages.

A second case for enzymatic replacement may occur in the cholesterol synthesis pathway from brown algae. The cholesterol biosynthesis pathway is known since the 1960’s to be highly variable in animals. The experimental characterization of these variations in multiple tissues and cell types has increased thanks to the advances in analytical techniques (Mitsche et al., 2015). This has also been helpful to revisit the data on plant and algal sterol biosynthesis pathways, for which high plasticity had already been hypothesized (Benveniste, 2004). A biosynthetic pathway from cycloartenol to cholesterol has been proposed for solanacean plants (Sawai et al., 2014; Sonawane et al., 2016) and has been used as a template to propose a similar pathway in red algae (Belcour et al., 2020). This is called the “early SSR’’ pathway on **Figure 3**, because of the early occurrence of sterol side-chain reduction. However, due to the evidence for desmosterol in red algae (**Figure 3**), a second pathway, called the late SSR pathway, was also proposed to accommodate those data. Independent studies on diatoms and brown algae then came to the conclusion that a late SSR pathway was existing in those organisms (Gallo et al., 2020; Girard et al., 2021). Although those pathways are lumped together in this article for clarity, they exhibit slight differences.

Regarding the late SSR pathway, a second case of enzymatic replacement may occur in brown algae. Indeed, despite the presence of desmosterol and cholesterol in many species, suggesting the existence of side chain reductase activity, no sequence corresponding to the canonical eukaryotic enzyme has been identified so far in brown algae (Girard et al., 2021). However in this case, the alternative enzyme, if existing, is completely unknown. Interestingly, the most abundant brown algal sterol is fucosterol, which does not necessitate such a side-chain reduction, so in this case the loss of the canonical enzyme may correlate with some kind of relaxation of the selection pressure. This may also explain the loss of C22 desaturase, canonically catalysed by a CYP710 enzyme, which has also been lost in brown algae (Teng et al., 2019). Regarding the inversion in enzyme order, the demonstration is still incomplete concerning side-chain reductases, given that biosynthesis enzymes between cycloartenol and cholesterol have not yet been biochemically characterized in macroalgae.

### Some changes in enzyme order in phosphatidyl-choline biosynthesis

**Figure 4:**
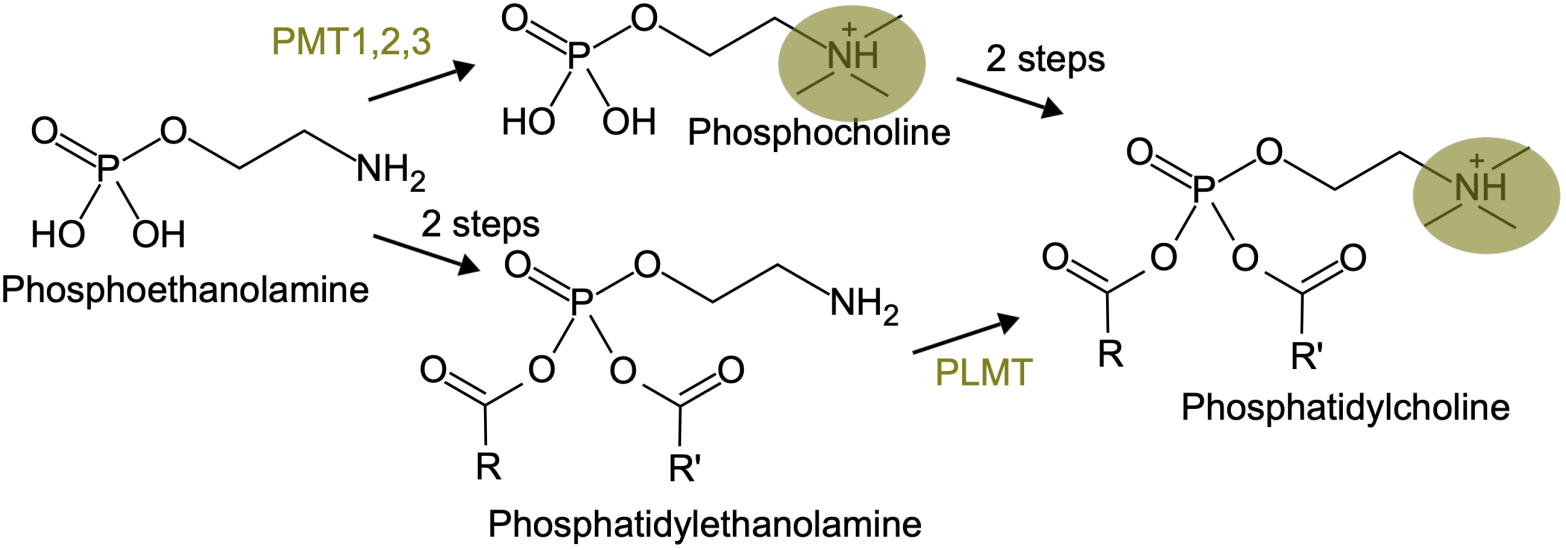
An inversion in enzyme order in the phosphatidyl-choline biosynthesis pathway. The three methylation steps, carried on either by PMT or PLMT, are highlighted in yellow.

One recently documented case of inversion in enzyme order is about the phosphatidylcholine biosynthesis pathway in plants (**Figure 4**). The classical pathway, described in vegetative tissues, involves phosphatidylcholine synthesis from phosphoethanolamine through a phosphocholine intermediate, that is produced by three successive methylations catalyzed by the phospho-base N-methyltransferases PMT1, 2 and 3. In reproductive tissues, phosphatidylcholine synthesis goes through a phosphatidyl-ethanolamine intermediate, with the methylation steps occurring at the end, catalyzed by a phospholipid N-methyltransferase (PLMT, Tan and Nakamura, 2022). This is reminiscent of the situation in animals, where phosphatidylcholine synthesis through phosphocholine is the pathway present in most tissues, whereas phosphatidylcholine synthesis through phosphatidyl-ethanolamine occurs in the liver.

### Biotic interactions and the origin of metabolic plasticity

The examples discussed above indicate that there is some empirical evidence, although not always fully characterized biochemically, for metabolic pathway drift in plant and algal lipid biosynthesis pathways. While the picture becomes clearer regarding the description of contemporaneous pathways, the question about the origin of changes is not always easy to address. In cases where a given pathway is widespread across eukaryotes, the term “alternative” suggests that the other one has appeared later on during evolution, leading to enzymatic replacement. However, regarding inversion in enzyme order, especially in pathways with multiple enzymes, it is much more difficult to infer the evolution of such changes, especially when pathways from highly diverging organisms are compared.

A second question is related to the way we interpret those variations in metabolic pathways. The term “drift” seems appropriate to remind that, especially over large evolutionary distances, variations have to be interpreted as mainly neutral (Haag and True, 2021). However, at a smaller timescale, and especially with pathways connected to signaling processes, one obvious factor that may significantly contribute to metabolic pathway variation is the various cross-regulations operated during species interactions. For example, fungal pathogens inactivate jasmonate signaling in their plant host by omega-hydroxylation (Patkar et al., 2015), or produce specific peptides, which are involved in infection through binding of polar heads from ceramides (Haslam and Feussner, 2022). Also, parasitic dinoflagellates from the *Amoebophrya* genus (order: Syndiniales) adjust their sterol membrane composition to the one from their host in the *Karlodinium* genus (order: Gymnodiniales), which enable them to avoid the detrimental effect of their own membrane-permeabilizing toxins (Place et al., 2009). Beyond examples from plant and algal lipids, it has also been hypothesized that metabolic plasticity on its own may provide selective advantages in experiments of artificial evolution among bacterial communities (Morris, 2015). More specifically, the ability to introduce variations in the metabolic pathways may allow a helper species to escape the dependence of the beneficiary species, as leaking products may change. This would be even more advantageous if beneficiary species adopt parasitic relationships with the helper.

## Is metabolic pathway drift the result of long-term selective processes linked with biotic interactions?

### Recently diverged biotic partners to study metabolic coevolution

**Figure 5:**
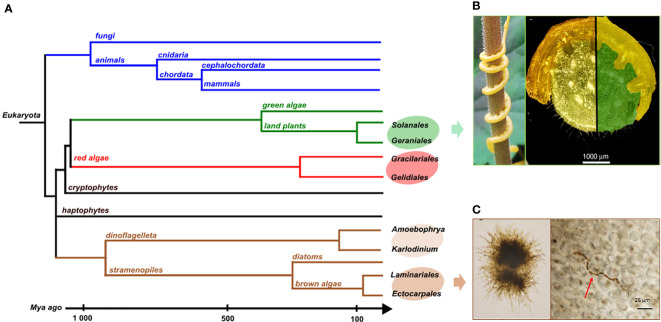
**(A)** Phylogenetic tree of the different groups mentioned here and their divergence times. Illustrations of biotic interactions in closely related orders: **(B)** Land plant lineage with the Solanale *Cuscuta campestris* infecting a Geraniale, *Pelargonium zonale* and a close-up view of parasitic stems infecting its host (pictures from Vogel et al., 2018); **(C)** Brown algal lineage with *Laminarionema elsbetiae*, an endophytic Ectocarpale isolated from *Saccharina latissima*, in unialgal culture (left) and growing in between the cells of its kelp host (right, picture from Bernard et al., 2018).

Many model systems for interactions between species involve organisms that have diverged a long time ago. For example, most of the eukaryotic endophytes in land plants belong to lineages that diverged a billion years ago from their plant hosts, such as fungi or oomycetes (Hardoim et al., 2015). In these cases, their biosynthetic pathways are the results of long-term evolution, making it difficult or even impossible to infer drift events or reconstruct common ancestral metabolic features. To study in detail the evolution of metabolic pathways, it is therefore necessary to focus on interacting species which are phylogenetically more closely related. These kinds of long-lasting biotic interactions have been described in land plant, red and brown alga lineages as shown on **Figure 5**.

### Gene losses related to parasitic interactions between land plants

Within angiosperms, there are some experimental systems to study gene loss related to parasitism with closely related hosts and parasites (Jhu and Sinha, 2022). Among them, the dodders, belonging to the *Cuscuta* genus, have a median divergence time with their closest hosts belonging to the Convolvulacea family, estimated around 57 Mya. They also parasitize other plants of agronomic importance among the Solanales which some belong to the Geraniales, as *Pelargonium zonale* (**Figure 5B**). In that case, the divergence times goes to around 118 Mya (Kumar et al., 2022). The rootless dodder plantlets gradually cover the plant via the stems to parasitize the stems and leaves of the host using suckers, or haustoria (**Figure 5B**), allowing them to access the vascular system of the host and thus take the essential elements for their development, without using photosynthetic activities. The dodder genome reveals specific gene losses linked to parasitism (Sun et al., 2018; Vogel et al., 2018). Among these, some have been identified as keys to photosynthesis, defense-related, leaf and root development or to the control of flowering time (Shen et al., 2020). Due to this biotic interaction, the host plant covers part of the needs of the parasitic plant which therefore no longer features some essential plant functions.

### Contrasting patterns of gene losses in red algae

However, in the case of other biotic interactions, an example in red algae demonstrates that there is no systematic gene loss in the parasitic partner. Indeed, an initial study from Preuss et al., 2020 on a New Zealand population of red algae reported a reduction in the plastid genome as part of an interaction between a parasite close to the Gracilariales and a host belonging to the Gelidiales. The divergence time between the order of Gracilariales and Gelidiales is 336 Mya (**Figure 5A**). Among the gene losses identified, all photosynthesis-related genes were shown to have been lost in the plastid genome. However, no such loss has been recovered in the mitochondrial genome which seems to be similar to what is expected for other free-living red algae. Nevertheless, in 2023, a second study carried out on red algae populations from the Ceramiales (**Figure 5A**) located in Australia and the Canary Islands showed no gene loss in genomes of species under the same conditions of biotic interaction. Yet, two pseudogenes were discovered in the plastid genome, implying a putative gene loss in the future (Preuss et al., 2023). As the parasitic red algae and their host are from the same *Laurencia* clade, the divergence time scale is smaller. It could explain why the loss of genes is not yet visible. Moreover, there are still free-living species in the same genus which could explain a selective pressure preventing the loss of genes.

### The Laminariales kelp/endophytic Ectocarpales interaction in brown algae

In brown algal lineage, hosts and endophyte species pairs offer unique model systems to address questions on metabolic coevolution. It enables the reconstruction of the establishment of interactions between metabolic pathways of both partners, going back to their closely-related common ancestor. The kelps *Saccharina latissima* and *Laminaria digitata* are two ecologically important brown algae belonging to Laminariales. In natural conditions, each of these two species harbors specific filamentous endophytes (**Figure 5C**), belonging to the group of Ectocarpales. *Laminarionema elsbetiae* is predominantly endophytic of *Saccharina latissima* which constitutes its natural host (Bernard et al., 2018). The presence of the filamentous Ectocarpales in *Laminaria digitata* cultures triggers the expression of genes likely involved in defense reactions (Xing et al., 2021). The early activation of these defense mechanisms could explain why in nature *Laminarionema elsbetiae* is barely detected inside *Laminaria digitata* (Bernard et al., 2019). Moreover, the phylogenetic proximity between kelps and their endophytes increases the probability that orthologous enzymes have identical catalytic activities, allowing compensation phenomena in the event of gene losses by endophytes. An additional advantage of this system is that the free-living *Ectocarpus* is already a model organism to study diverse aspects of brown algal biology. In fact, several features found in the members of the Ectocarpales makes them well adapted for genetic studies such as short life cycles, small size at maturity and small genome sizes (Badis et al., 2021). In addition, the availability of reverse genetic tools, such as CRISPR-Cas9 mutagenesis, greatly enhances the utility of *Ectocarpus* as a model organism for investigating the phenotypes of brown algae that are relevant to metabolic pathway function and evolution.

## Perspective: towards a general framework about metabolic pathway plasticity and evolution

Apart from enzyme replacement and changes in enzyme order, there are several other additional possible mechanisms leading to metabolic pathway drift such as gene expression increase instead of gene duplication (Xu et al., 2017), variation in subcellular localization (Río Bártulos et al., 2018; Conart et al., 2023), changes in rate-limiting enzymes (Orlenko et al., 2016) or regulatory plasticity (Coton et al., 2022; Coton et al., 2023). However, before going into exploration of other possible mechanisms, it is necessary to fully understand how enzyme replacement and changes in enzyme order occur. The metabolic drift by enzyme replacement supposes that an original enzyme in a given pathway can be substituted by a new, potentially unrelated, one. In the following section we explore current hypotheses related to the selective advantages of gene losses and gains, and we propose putative mechanisms leading to enzyme replacement and change in enzyme order.

In Bacteria, horizontal gene transfers (HGT) are considered as the principal way to acquire new genes. The acquired gene may provide adaptive advantages, participating in the ecological adaptation, response to stress, drug resistance. In these cases, the transferred genes are fixed in the populations, increasing the size of their pangenomes (Brockhurst et al., 2019). Some species are particularly prone to acquire a wide diversity of transferred genes, their genomes are said to be “open”, and new sequenced genomes will continuously reveal new rare genes. An opposing mechanism consists in losing genes instead, which may also provide adaptive advantages. This is referred to as the Black Queen Hypothesis, BQH (Morris et al., 2012; Morris, 2015). In ecologically stable environments, organisms denoted as “beneficiaries” lose functions/products that are still provided by other organisms, the “helpers”. Becoming a beneficiary species decreases the energetic cost of producing the lost function, hence providing a selective advantage. Both phenomena, genome openness to HGT and BQH, could also be neutral, owing to the fact that concerned populations have small effective sizes. In this case there are no particular selective advantages in gaining or losing genes. Global trends for gene loss or gain suggest possible interplay allowing metabolic drift by enzyme replacement to occur, a lost gene being subsequently replaced by a new one. A first hypothesis for such a scenario requires the possibility to benefit from a pool of available enzymes functionally suitable for replacement. While HGT appears predominant in bacteria, paralogous duplication and sub-functionalization are sources of gene and function gains in Eukaryota (Force et al., 1999). Enzymes may have promiscuous enzymatic functions, involving interactions with a main substrate and also with accessory substrates (Peracchi, 2018). Unrelated or paralogous enzymes that enable degradation of a common substrate can furnish candidates for enzyme replacement. Pathways can also be recomposed though intracellular leakage of labile metabolic intermediates, which can restore metabolic function under strong selection pressure (Medina-Carmona et al., 2023).

**Figure 6:**
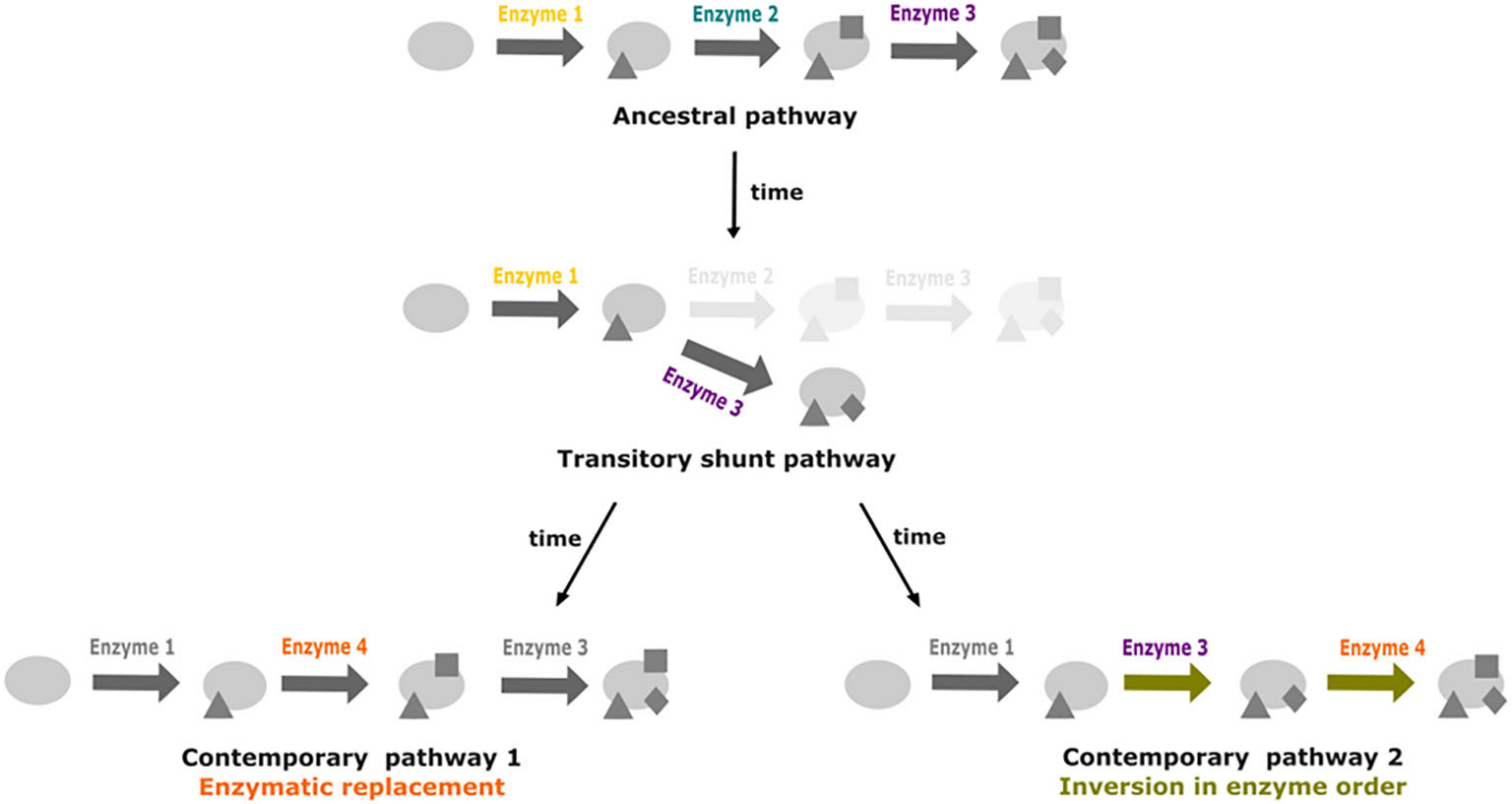
Promiscuity and shunt pathways as facilitators for metabolic pathway drift. Starting from a three step ancestral pathway, the loss of the enzyme 2 can lead to the formation of a shunt pathway if promiscuity of enzyme 3 enables it to act directly on the product of reaction catalyzed by enzyme 1. Recovery of the activity from enzyme 2 can occur through replacement by enzyme 4. In a second case, insertion of enzyme 4 at the end of the pathway could lead to inversion in enzyme order for the last two steps of the pathway.

A second hypothesis would involve a possible trade-off between becoming a beneficiary with some reactions lost, or recovering them back. HGT, from bacterial symbionts or from the holobiont, or metabolic rewiring from paralogs or promiscuous enzymatic activities (**Figure 6**), may recover the functions when auxotrophy becomes too costly.

On the one hand relying on other organisms to perform specific functions can compensate for gene loss, but such compensation introduces new constraints related to the presence of the required organism and the exchange of the required metabolites. These constraints can have a negative impact on the possibility of adaptation of interdependent organisms to stress phenomenon. It has been observed that auxotrophic strains of *E. coli* lose their mutualistic interactions and regain metabolic autonomy when exposed to increasing antibiotic stresses (Pauli et al., 2022). These results illustrate how regaining the lost function and autonomy provides adaptive advantage. Thus, evading auxotrophy by function regain may participate in the observed metabolic plasticity.

All the examples we discussed in this review could obviously become strengthened by additional experimental data. However, we hope we convincingly show that there is already enough data to fuel general thinking about metabolic pathway evolution, and that our paper will stimulate further integration between theoretical and empirical work.

## Author contributions

MZ: Writing – original draft, Writing – review & editing. AB: Writing – review & editing, Writing – original draft. LD: Writing – review & editing. AS: Writing – review & editing. SB: Writing – review & editing, Writing – original draft. CL: Writing – review & editing. GM: Writing – original draft, Writing – review & editing.

## References

[B1] Badis Y. Scornet D. Harada M. Caillard C. Godfroy O. Raphalen M.. (2021). Targeted CRISPR-Cas9-based gene knockouts in the model brown alga *Ectocarpus* . New Phytol. 231, 2077–2091. doi: 10.1111/nph.17525 34076889

[B2] Belcour A. Girard J. Aite M. Delage L. Trottier C. Marteau C.. (2020). Inferring biochemical reactions and metabolite structures to understand metabolic pathway drift. iScience 23, 100849. doi: 10.1016/j.isci.2020.100849 32058961 PMC6997860

[B3] Benveniste P. (2004). Biosynthesis and accumulation of sterols. Annu. Rev. Plant Biol. 55, 429–457. doi: 10.1146/annurev.arplant.55.031903.141616 15377227

[B4] Bernard M. Rousvoal S. Jacquemin B. Ballenghien M. Peters A. F. Leblanc C. (2018). qPCR-based relative quantification of the brown algal endophyte *Laminarionema elsbetiae* in *Saccharina latissima*: variation and dynamics of host—endophyte interactions. J. Appl. Phycol. 30, 2901–2911. doi: 10.1007/s10811-017-1367-0 30416259 PMC6208874

[B5] Bernard M. S. Strittmatter M. Murúa P. Heesch S. Cho G. Y. Leblanc C.. (2019). Diversity, biogeography and host specificity of kelp endophytes with a focus on the genera *Laminarionema* and *Laminariocolax* (Ectocarpales, Phaeophyceae). Eur. J. Phycol. 54, 39–51. doi: 10.1080/09670262.2018.1502816

[B6] Blée E. (2002). Impact of phyto-oxylipins in plant defense. Trends Plant Sci. 7, 315–322. doi: 10.1016/S1360-1385(02)02290-2 12119169

[B7] Brockhurst M. A. Harrison E. Hall J. P. J. Richards T. McNally A. MacLean C. (2019). The ecology and evolution of pangenomes. Curr. Biol. 29, R1094–R1103. doi: 10.1016/j.cub.2019.08.012 31639358

[B8] Brodhun F. Feussner I. (2011). Oxylipins in fungi. FEBS J. 278, 1047–1063. doi: 10.1111/j.1742-4658.2011.08027.x 21281447

[B9] Cannon A. E. Chapman K. D. (2021). Lipid signaling through G proteins. Trends Plant Sci. 26, 720–728. doi: 10.1016/j.tplants.2020.12.012 33468433

[B10] Chang M. S. Boeglin W. E. Guengerich F. P. Brash A. R. (1996). Cytochrome P450-dependent transformations of 15 R-and 15 S-hydroperoxyeicosatetraenoic acids: stereoselective formation of epoxy alcohol products. Biochemistry 35, 464–471. doi: 10.1021/bi952081v 8555216

[B11] Conart C. Bomzan D. P. Huang X. Q. Bassard J. E. Paramita S. N. Saint-Marcoux D.. (2023). A cytosolic bifunctional geranyl/farnesyl diphosphate synthase provides MVA-derived GPP for geraniol biosynthesis in rose flowers. Proc. Natl. Acad. Sci. U.S.A. 120, e2221440120. doi: 10.1073/pnas.2221440120 37126706 PMC10175749

[B12] Coton C. Dillmann C. de Vienne D. (2023). Evolution of enzyme levels in metabolic pathways: A theoretical approach. Part 2. J. Theor. Biol. 558, 111354. doi: 10.1016/j.jtbi.2022.111354 36427531

[B13] Coton C. Talbot G. Le Louarn M. Dillmann C. de Vienne D. (2022). Evolution of enzyme levels in metabolic pathways: A theoretical approach. Part 1. J. Theor. Biol. 538, 111015. doi: 10.1016/j.jtbi.2022.111015 35016894

[B14] Fontana A. d’Ippolito G. Cutignano A. Romano G. Lamari N. Massa Gallucci A.. (2007). LOX-induced lipid peroxidation mechanism responsible for the detrimental effect of marine diatoms on zooplankton grazers. Chembiochem 8, 1810–1818. doi: 10.1002/cbic.200700269 17886321

[B15] Force A. Lynch M. Pickett F. B. Amores A. Yan Y. L. Postlethwait J. (1999). Preservation of duplicate genes by complementary, degenerative mutations. Genetics 151, 1531–1545. doi: 10.1093/genetics/151.4.1531 10101175 PMC1460548

[B16] Gallo C. Landi S. d’Ippolito G. Nuzzo G. Manzo E. Sardo A.. (2020). Diatoms synthesize sterols by inclusion of animal and fungal genes in the plant pathway. Sci. Rep. 10, 4204. doi: 10.1038/s41598-020-60993-5 32144288 PMC7060231

[B17] Gerwick W. H. Moghaddam M. Hamberg M. (1991). Oxylipin metabolism in the red alga *Gracilariopsis lemaneiformis*: mechanism of formation of vicinal dihydroxy fatty acids. Arch. Biochem. Biophys. 290, 436–444. doi: 10.1016/0003-9861(91)90563-X 1929410

[B18] Girard J. Lanneau G. Delage L. Leroux C. Belcour A. Got J.. (2021). Semi-quantitative targeted gas chromatography-mass spectrometry profiling supports a late side-chain reductase cycloartenol-to-cholesterol biosynthesis pathway in brown algae. Front. Plant Sci. 12. doi: 10.3389/fpls.2021.648426 PMC8112355 33986764

[B19] Haag E. S. True J. R. (2021). “Developmental system drift,” in Evolutionary developmental biology. Eds. Nuño de la Rosa L. Müller G. B. (Cham: Springer), 99–110. doi: 10.1007/978-3-319-32979-6_83

[B20] Hardoim P. R. van Overbeek L. S. Berg G. Pirttilä A. M. Compant S. Campisano A.. (2015). The hidden world within plants: ecological and evolutionary considerations for defining functioning of microbial endophytes. Microbiol. Mol. Biol. Rev. 79, 293–320. doi: 10.1128/mmbr.00050-14 26136581 PMC4488371

[B21] Hart K. M. Harms M. J. Schmid B. H. Elya C. Thornton J. W. Marqusee S. (2014). Thermodynamic system drift in protein evolution. PloS Biol. 12, e1001994. doi: 10.1371/journal.pbio.1001994 25386647 PMC4227636

[B22] Haslam T. M. Feussner I. (2022). Diversity in sphingolipid metabolism across land plants. J. Exp. Bot. 73, 2785–2798. doi: 10.1093/jxb/erab558 35560193 PMC9113257

[B23] Jhu M. Y. Sinha N. R. (2022). Parasitic plants: an overview of mechanisms by which plants perceive and respond to parasites. Annu. Rev. Plant Biol. 73, 433–455. doi: 10.1146/annurev-arplant-102820-100635 35363532

[B24] Kumar S. Suleski M. Craig J. M. Kasprowicz A. E. Sanderford M. Li M.. (2022). TimeTree 5: an expanded resource for species divergence times. Mol. Biol. Evol. 39, msac174. doi: 10.1093/molbev/msac174 35932227 PMC9400175

[B25] Lee D. S. Nioche P. Hamberg M. Raman C. S. (2008). Structural insights into the evolutionary paths of oxylipin biosynthetic enzymes. Nature 455, 363–368. doi: 10.1038/nature07307 18716621

[B26] Mashhadi Z. Newcomer M. E. Brash A. R. (2016). The thr-his connection on the distal heme of catalase-related hemoproteins: A hallmark of reaction with fatty acid hydroperoxides. Chembiochem 17, 2000–2006. doi: 10.1002/cbic.201600345 27653176 PMC5267355

[B27] Medina-Carmona E. Gutierrez-Rus L. I. Manssour-Triedo F. Newton M. S. Gamiz-Arco G. Mota A. J.. (2023). Cell survival enabled by leakage of a labile metabolic intermediate. Mol. Biol. Evol. 40, msad032. doi: 10.1093/molbev/msad032 36788592 PMC9989741

[B28] Mitsche M. A. McDonald J. G. Hobbs H. H. Cohen J. C. (2015). Flux analysis of cholesterol biosynthesis in *vivo* reveals multiple tissue and cell-type specific pathways. Elife 4, e07999. doi: 10.7554/eLife.07999 26114596 PMC4501332

[B29] Morris J. J. (2015). Black Queen evolution: the role of leakiness in structuring microbial communities. Trends Genet. 31, 475–482. doi: 10.1016/j.tig.2015.05.004 26078099

[B30] Morris J. J. Lenski R. E. Zinser E. R. (2012). The Black Queen Hypothesis: evolution of dependencies through adaptive gene loss. mBio 3, e00036–e00012. doi: 10.1128/mBio.00036-12 22448042 PMC3315703

[B31] Nelson D. Werck-Reichhart D. (2011). A P450-centric view of plant evolution. Plant J. 66, 194–211. doi: 10.1111/j.1365-313X.2011.04529.x 21443632

[B32] Orlenko A. Hermansen R. A. Liberles D. A. (2016). Flux control in glycolysis varies across the tree of life. J. Mol. Evol. 82, 146–161. doi: 10.1007/s00239-016-9731-2 26920685

[B33] Palsson A. Steele S. E. (2023). Adaptive cellular evolution or cellular system drift in hares. Mol. Ecol. 32, 4093–4096. doi: 10.1111/mec.17030 37259276

[B34] Patkar R. N. Benke P. I. Qu Z. Chen Y. Y. Yang F. Swarup S.. (2015). A fungal monooxygenase-derived jasmonate attenuates host innate immunity. Nat. Chem. Biol. 11, 733–740. doi: 10.1038/nchembio.1885 26258762

[B35] Pauli B. Oña L. Hermann M. Kost C. (2022). Obligate mutualistic cooperation limits evolvability. Nat. Commun. 13, 337. doi: 10.1038/s41467-021-27630-9 35039522 PMC8764027

[B36] Peracchi A. (2018). The limits of enzyme specificity and the evolution of metabolism. Trends Biochem. Sci. 43, 984–996. doi: 10.1016/j.tibs.2018.09.015 30472990

[B37] Place A. R. Bai X. Kim S. Sengco M. R. Wayne Coats D. (2009). Dinoflagellate host-parasite sterol profiles dictate karlotoxin sensitivity. J. Phycol. 45, 375–385. doi: 10.1111/j.1529-8817.2009.00649.x 27033816

[B38] Pollier J. Vancaester E. Kuzhiumparambil U. Vickers C. E. Vandepoele K. Goossens A.. (2019). A widespread alternative squalene epoxidase participates in eukaryote steroid biosynthesis. Nat. Microbiol. 4, 226–233. doi: 10.1038/s41564-018-0305-5 30478288

[B39] Preuss M. Díaz-Tapia P. Verbruggen H. Zuccarello G. C. (2023). Gene-rich plastid genomes of two parasitic red algal species, Laurencia australis and L. verruciformis (Rhodomelaceae, Ceramiales), and a taxonomic revision of Janczewskia. J. Phycol 59, 950–962. doi: 10.1111/jpy.13373 37638497

[B40] Preuss M. Verbruggen H. Zuccarello G. C. (2020). The organelle genomes in the photosynthetic red algal parasite pterocladiophila hemisphaerica (Florideophyceae, rhodophyta) have elevated substitution rates and extreme gene loss in the plastid genome. J. Phycol. 56, 1006–1018. doi: 10.1111/jpy.12996 32215918

[B41] Río Bártulos C. Rogers M. B. Williams T. A. Gentekaki E. Brinkmann H. Cerff R.. (2018). Mitochondrial glycolysis in a major lineage of eukaryotes. Gen. Biol. Evol. 10, 2310–2325. doi: 10.1093/gbe/evy164 PMC6198282 30060189

[B42] Ruocco N. Albarano L. Esposito R. Zupo V. Costantini M. Ianora A. (2020). Multiple roles of diatom-derived oxylipins within marine environments and their potential biotechnological applications. Mar. Drugs 18, 342. doi: 10.3390/md18070342 32629777 PMC7401250

[B43] Sabatino V. Orefice I. Marotta P. Ambrosino L. Chiusano M. L. d'Ippolito G.. (2022). Silencing of a *Pseudo-nitzschia arenysensis* lipoxygenase transcript leads to reduced oxylipin production and impaired growth. New Phytol. 233, 809–822. doi: 10.1111/nph.17739 34533849

[B44] Sawai S. Ohyama K. Yasumoto S. Seki H. Sakuma T. Yamamoto T.. (2014). Sterol side chain reductase 2 is a key enzyme in the biosynthesis of cholesterol, the common precursor of toxic steroidal glycoalkaloids in potato. Plant Cell 26, 3763–3774. doi: 10.1105/tpc.114.130096 25217510 PMC4213163

[B45] Sémon M. Steklikova K. Mouginot M. Peltier M. Veber P. Guéguen L.. (2020). Phenotypic innovation in one tooth induced concerted developmental evolution in another. bioRxiv. 2020 doi: 10.1101/2020.04.22.043422

[B46] Shen G. Liu N. Zhang J. Xu Y. Baldwin I. T. Wu J. (2020). *Cuscuta australis* (dodder) parasite eavesdrops on the host plants’ FT signals to flower. Proc. Natl. Acad. Sci. U.S.A. 117, 23125–23130. doi: 10.1073/pnas.2009445117 32868415 PMC7502711

[B47] Sonawane P. D. Pollier J. Panda S. Szymanski J. Massalha H. Yona M.. (2016). Plant cholesterol biosynthetic pathway overlaps with phytosterol metabolism. Nat. Plants 3, 16205. doi: 10.1038/nplants.2016.205 28005066

[B48] Sun G. Xu Y. Liu H. Sun T. Zhang J. Hettenhausen C.. (2018). Large-scale gene losses underlie the genome evolution of parasitic plant *Cuscuta australis* . Nat. Commun. 9, 2683. doi: 10.1038/s41467-018-04721-8 29992948 PMC6041341

[B49] Tan Y. R. Nakamura Y. (2022). The importance of Arabidopsis PHOSPHOLIPID N-METHYLTRANSFERASE in glycerolipid metabolism and plant growth. J. Exp. Bot. 73, 2971–2984. doi: 10.1093/jxb/erac049 35560202

[B50] Teng L. Fan X. Nelson D. R. Han W. Zhang X. Xu D.. (2019). Diversity and evolution of cytochromes P450 in stramenopiles. Planta 249, 647–661. doi: 10.1007/s00425-018-3028-1 30341489

[B51] Toporkova Y. Y. Fatykhova V. S. Gogolev Y. V. Khairutdinov B. I. Mukhtarova L. S. Grechkin A. N. (2017). Epoxyalcohol synthase of *Ectocarpus siliculosus.* First CYP74-related enzyme of oxylipin biosynthesis in brown algae. Biochim. Biophys. Acta Mol. Cell Biol. Lipids 1862, 167–175. doi: 10.1016/j.bbalip.2016.11.007 27863255

[B52] Toporkova Y. Y. Smirnova E. O. Mukhtarova L. S. Grechkin A. N. (2022). Lipoxygenase pathway in brown algae: The biosynthesis of novel oxylipins ‘ectocarpins’ by hydroperoxide bicyclase CYP5164A3 of *Ectocarpus siliculosus* . Biochim. Biophys. Acta Mol. Cell Biol. Lipids 1867, 159205. doi: 10.1016/j.bbalip.2022.159205 35835431

[B53] True J. R. Haag E. S. (2001). Developmental system drift and flexibility in evolutionary trajectories. Evol. Dev. 3, 109–119. doi: 10.1046/j.1525-142x.2001.003002109.x 11341673

[B54] Turner J. G. Ellis C. Devoto A. (2002). The jasmonate signal pathway. Plant Cell 14, S153–S164. doi: 10.1105/tpc.000679 12045275 PMC151253

[B55] Vogel A. Schwacke R. Denton A. K. Usadel B. Hollmann J. Fischer K.. (2018). Footprints of parasitism in the genome of the parasitic flowering plant *Cuscuta campestris* . Nat. Commun. 9, 2515. doi: 10.1038/s41467-018-04344-z 29955043 PMC6023873

[B56] Wasternack C. Feussner I. (2018). The oxylipin pathways: biochemistry and function. Annu. Rev. Plant Biol. 69, 363–386. doi: 10.1146/annurev-arplant-042817-040440 29166128

[B57] Xing Q. Bernard M. Rousvoal S. Corre E. Markov G. V. Peters A. F.. (2021). Different early responses of laminariales to an endophytic infection provide insights about kelp host specificity. Front. Mar. Sci. 8. doi: 10.3389/fmars.2021.742469

[B58] Xu S. Brockmöller T. Navarro-Quezada A. Kuhl H. Gase K. Ling Z.. (2017). Wild tobacco genomes reveal the evolution of nicotine biosynthesis. Proc. Natl. Acad. Sci. U.S.A. 114, 6133–6138. doi: 10.1073/pnas.1700073114 28536194 PMC5468653

